# Benchmarking Accelerometer and CNN-Based Vision Systems for Sleep Posture Classification in Healthcare Applications

**DOI:** 10.3390/s25123816

**Published:** 2025-06-18

**Authors:** Minh Long Hoang, Guido Matrella, Dalila Giannetto, Paolo Craparo, Paolo Ciampolini

**Affiliations:** Department of Engineering and Architecture, University of Parma, 43124 Parma, Italy; guido.matrella@unipr.it (G.M.); dalila.giannetto@studenti.unipr.it (D.G.); paolo.craparo@studenti.unipr.it (P.C.); paolo.ciampolini@unipr.it (P.C.)

**Keywords:** accelerometer, deep neural network, vision-based system, sleep posture classification, CNN

## Abstract

Sleep position recognition plays a crucial role in diagnosing and managing various health conditions, such as sleep apnea, pressure ulcers, and musculoskeletal disorders. Accurate monitoring of body posture during sleep can provide valuable insights for clinicians and support the development of intelligent healthcare systems. This research presents a comparative analysis of sleep position recognition using two distinct approaches: image-based deep learning and accelerometer-based classification. There are five classes: prone, supine, right side, left side, and wake up. For the image-based method, the Visual Geometry Group 16 (VGG16) convolutional neural network was fine-tuned with data augmentation strategies including rotation, reflection, scaling, and translation to enhance model generalization. The image-based model achieved an overall accuracy of 93.49%, with perfect precision and recall for “right side” and “wakeup” positions, but slightly lower performance for “left side” and “supine” classes. In contrast, the accelerometer-based method employed a feedforward neural network trained on features extracted from segmented accelerometer data, such as signal sum, standard deviation, maximum, and spike count. This method yielded superior performance, reaching an accuracy exceeding 99.8% across most sleep positions. The “wake up” position was particularly easy to detect due to the absence of body movements such as heartbeat or respiration when the person is no longer in bed. The results demonstrate that while image-based models are effective, accelerometer-based classification offers higher precision and robustness, particularly in real-time and privacy-sensitive scenarios. Further comparisons of the system characteristics, data size, and training time are also carried out to offer crucial insights for selecting the appropriate technology in clinical, in-home, or embedded healthcare monitoring applications.

## 1. Introduction

Sleep posture is a critical factor influencing various health conditions, including sleep apnea, pressure ulcers, and musculoskeletal disorders. Improper or sustained sleeping positions are strongly associated with a range of medical issues, including obstructive sleep apnea, gastroesophageal reflux, musculoskeletal pain, and the development of pressure ulcers in bedridden patients. Supine sleeping is known to exacerbate apnea events [1], while side-sleeping may reduce snoring and improve breathing in individuals with respiratory disorders. In nursing care, continuous monitoring of sleep posture is essential for the early detection of pressure sore risks [2]. Practically, accurate monitoring of sleep positions can aid in diagnosing sleep-related issues and improving patient care. In addition, sleep position is an important parameter in neurological disorders such as epilepsy and Parkinson’s disease [3], where involuntary nocturnal movements or abnormal postures can serve as early indicators of disease progression [4]. Recent work by Chi et al. [5] found that sleep deprivation significantly alters muscle–brain connectivity during isometric contractions, highlighting the systemic impact of poor sleep quality and posture on the sensorimotor network.

For elderly populations, fall risk and posture instability during sleep are also areas of concern that require effective and non-intrusive monitoring [6,7]. In clinical care, especially for immobile or elderly patients, continuous monitoring of sleep posture is vital for the early detection of pressure ulcer risks and fall prevention. Peruzzi et al. [8] emphasized the feasibility of embedded machine learning using sparse pressure sensor arrays for posture recognition, demonstrating how low-complexity systems can enable cost-effective, scalable solutions. Furthermore, abnormal or involuntary postures during sleep are increasingly recognized as early indicators of neurological conditions. For example, posture anomalies have been linked to disease progression in Parkinson’s disease and epilepsy, where sleep behavior often deviates from normative patterns [9]. Abdulsadig et al. [10] proposed a neck-mounted accelerometer system to detect sleep postures, offering a minimally invasive approach particularly relevant for disorders with nocturnal motor symptoms.

Despite the significant advancements in sleep posture recognition technologies, a direct and systematic comparison between image-based and accelerometer-based modalities [11,12,13] remains scarce in the literature. Most existing studies tend to focus exclusively on either visual or inertial data, often under specific conditions or application domains, which limits the generalizability of their findings. Therefore, this work aims to address this gap by conducting a thorough evaluation and head-to-head comparison of image-based deep learning and accelerometer-based machine learning methods for classifying five common sleep positions: supine, prone, left side, right side, and wake-up. The image-based classification approach employs a customized Visual Geometry Group 16 (VGG16) [14,15,16,17,18,19] convolutional neural network architecture, pre-trained on ImageNet and fine-tuned using a domain-specific dataset. To enhance robustness and improve model generalization to real-world conditions, extensive data augmentation techniques such as rotation, reflection, scaling, brightness adjustment, and translation are applied.

On the other hand, the accelerometer-based classification pipeline utilizes a lightweight feedforward neural network [20,21,22,23] trained on statistical and signal-based features extracted from raw tri-axial accelerometer data. These features include the sum of signal magnitude, standard deviation, maximum value, mean threshold crossing count, and spike count over fixed-length signal segments (corresponding to data acquisition frequency in one-second intervals). This segmentation captures micro-movements and postural dynamics that are indicative of different sleep positions.

While numerous studies have addressed sleep posture recognition using either image-based or accelerometer-based approaches, few have directly compared these two modalities under consistent conditions. For instance, Phan et al. [24] and Piriyajitakonkij et al. [25] advanced the field through deep learning architectures using signal and radar data, respectively, but their evaluations are confined to single modalities and specific sensing setups. Similarly, studies such as [26,27,28] proposed accelerometer-based or pressure-based systems, achieving notable performance, but without benchmarking them against vision-based alternatives. Another study [29] proposed a spatiotemporal CNN using a limited set of piezoelectric ceramic sensors, achieving high accuracy in posture detection. Conversely, vision-based solutions like those in [30,31,32] demonstrated high classification accuracy under controlled lighting but did not assess portability or privacy-preserving aspects.

This work distinguishes itself by conducting a direct, systematic comparison of two leading but typically siloed modalities: image-based and accelerometer-based classification for sleep posture recognition. Unlike prior studies that focused on modality-specific optimizations, we evaluate both under the same dataset conditions and performance metrics, allowing for a fair and comprehensive assessment. Furthermore, the proposed image-based model is fine-tuned using domain-specific data and augmented with robust pre-processing techniques to simulate real-world scenarios, while the accelerometer-based pipeline uses lightweight, interpretable features suitable for resource-constrained wearable systems.

Practically, this article contributes advanced research to health science as follows:

- The gap between vision and inertial sensing modalities is bridged through comparative benchmarking.
- Insights into practical trade-offs is analyzed, such as interpretability, intrusiveness, and robustness that are often overlooked in modality-specific research.
- Recommendations for modality selection or hybrid system design are provided, based on application constraints, such as home care, hospital monitoring, or elderly assistance.
- By comparing these two complementary modalities under consistent conditions with accuracy, confusion matrices, and performance metrics such as precision, recall, and F1-score, this study provides critical insights into their relative strengths. For instance, image-based models offer high spatial resolution and visual interpretability but require consistent lighting and unobstructed views, making them suitable for controlled environments. In contrast, accelerometer-based systems are privacy-preserving, unobtrusive, and cost-effective, well-suited for wearable or in-bed monitoring in home or clinical settings.

The findings of this comparative analysis aim to guide researchers and developers in selecting or integrating the most appropriate sensing modality based on application-specific constraints, thereby advancing the development of intelligent, non-invasive, and adaptive sleep posture monitoring systems. This paper is organized as follows: the first part describes the dataset, setup, and algorithms of the concerned methods. The second part is about experimental results and analysis. The final sections are about the conclusion and future work.

## 2. Materials and Methods

### 2.1. Dataset

There are 13 volunteers who participated in the data collection. Their ages are in the range of 24 to 35 years old, including both men and women. Their health was under normal conditions. The data of 11 people were used for training and the rest were used for tests. The accelerometer was fixed at the center of the bed frame, and volunteers were allowed to lie naturally in each posture without being restricted to a specific horizontal position. The system does not rely on the subject’s proximity to the sensor but instead classifies posture based on vibration patterns and movement features extracted from tri-axial accelerometer data. Importantly, physiological micro-vibrations such as respiration and heartbeat propagate differently depending on body orientation. Due to the heart’s anatomical position on the left side, the distinguishable signal characteristics are different between the left-side and right-side postures. These differences are captured in the features used by the model, enabling accurate classification even with moderate lateral displacement on the bed.

For the accelerometer-based method, 1,338,480 numerical samples were obtained with the acquisition frequency of 143 Hz, providing enough information for human body movement. Compared to higher rates, 143 Hz reduces data volume and energy consumption, which is crucial for long-term monitoring, especially in wearable or battery-operated systems. Meanwhile, 3445 images were taken for the image-based method, including five positions: supine, prone, left side, right side, and wake up. In the dataset, 70% was used for training, 10% for validation, and 20% for test.

#### 2.1.1. Camera-Based Method

**Figure 1:**
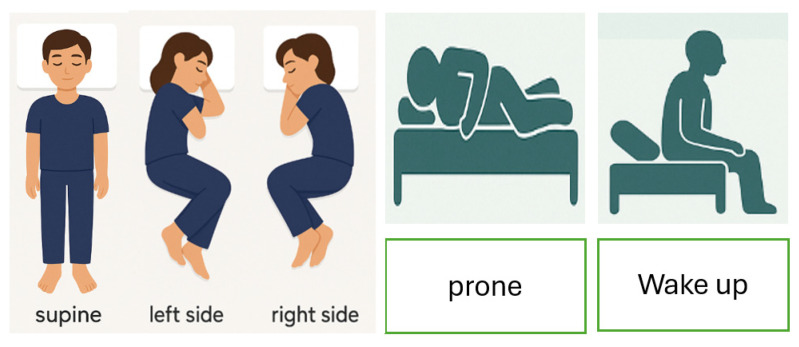
Sleep posture classification.

For the image classification, a series of images was captured from various angles for each sleep position as illustrated in Figure 1. The camera has 12 megapixels (MPs), an aperture of ƒ/1.8, focal length of 26 mm equivalent. To enhance the model’s generalization ability and robustness to variations in sleep posture images, a set of data augmentation techniques was applied. These augmentations simulate real-world variability in pose, scale, orientation, and position. There are four applied transformations as below:

- Random Rotation: Each input image is randomly rotated within a range of −15 to +15 degrees, which helps the model become invariant to slight angular variations in body posture. A range of ±15° maintains the semantic integrity of the posture (e.g., “supine” rotated 10° is still clearly “supine”). Larger rotations could distort the posture enough to confuse the label (e.g., a 90° rotation could make “left side” look like “supine”).
- Random X and Y Reflection: Images are randomly flipped horizontally and vertically. This simulates mirrored sleep positions such as switching from left to right side and improves robustness to sensor or camera orientation.
- Random Scaling: Each image is scaled by a random factor between 0.1× and 3× its original size which accounts for differences in camera zoom or distance from the subject.
- Random Translation in X and Y: Images are randomly shifted up to ±15 pixels horizontally and vertically. This process allows the model to handle positional shifts of the person in the bed or frame.

#### 2.1.2. Accelerometer-Based Method

**Figure 2:**
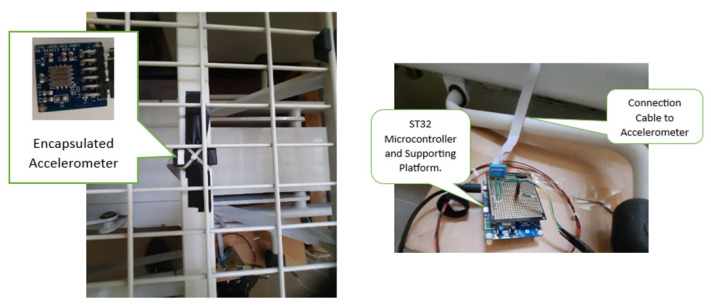
Encapsulated accelerometer and MCU platform under the bed frame.

**Figure 3:**
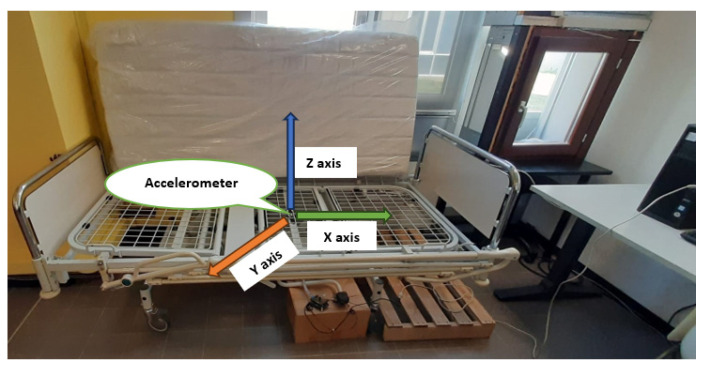
Smart bed under test.

The MEMS accelerometer ADXL355 [33] is concealed within a protective enclosure and positioned accurately beneath the bed frame, as shown in Figure 2, and it is connected to the STM32 embedded system platform [34]. This non-invasive method provides comfort for the user since the accelerometer is mounted under the bed which is not wearable in this case (Figure 3).

All acceleration values are converted to absolute value. From each 143-sample segment of each axis, the following five features are generated:

1. Sum of segment values represents the total accumulated acceleration like energy-like measure. Different postures involve varying amounts of movement and body orientation relative to gravity.
2. Standard deviation captures the variability or spreads of values in the segment. The variability of acceleration differs by posture. In supine or prone, movement is generally less variable as the body is more stable. Side posture usually has more variability due to how the limbs and torso interact with the bed surface or slight adjustments made to maintain comfort.
3. Count above the global mean number of samples in the segment that are above the mean of the full signal, indicating activity level. This feature helps distinguish postures by measuring how often acceleration exceeds a typical baseline.
4. Maximum value in the segment indicates the peak acceleration during that segment.
5. Spike count indicates the number of values in the segment that exceeds 10% of the global signal range which is the difference between the maximum and minimum value among these 143 samples. This feature captures sharp changes or movements such as twitching. Sharp accelerations indicate micro-movements or twitches that vary by posture.

By this pre-processing technique, there are a total of 15 input features from three axes (X, Y, Z) for the deep learning model, which enhances the output performance.

### 2.2. VGG16

**Figure 4:**
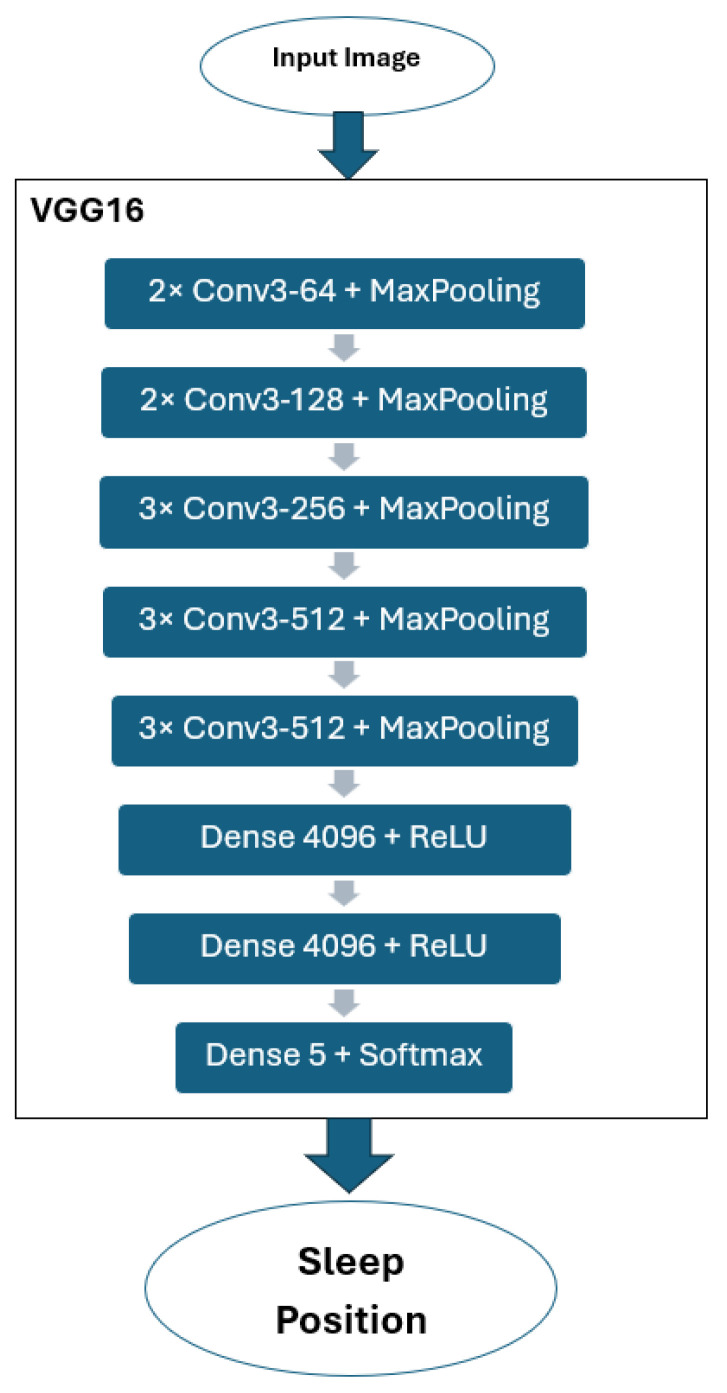
VGG16 Architecture.

VGG16 is a typical deep Convolutional Neural Network (CNN) design with numerous layers, having 16 weight layers: 13 convolutional layers + three fully connected layers) as demonstrated in Table 1 and Figure 4. In VGG16, the convolutional layers extract local features like edges, textures, and patterns from the input image. To capture fine details, 3 × 3 kernels are utilized. Then, the depth increases from 64 to 512 to learn more complex features. The max pooling layers reduce the spatial dimensions (height and width) while preserving the most important features. This process reduces computation and control overfitting. The fully connected layers flatten the 3D feature maps into 1D vectors, acting as a classifier using learned high-level features. The first two layers have 4096 neurons; the last layer has neurons equal to the number of classes. Softmax activation converts raw scores into probabilities over class labels.

Training progress, including loss and accuracy, is visualized in real-time using the training-progress plot. This setup provides a robust and efficient framework for classifying sleep postures from image data.

Where:

- Conv3-64 means a 3 × 3 convolution with 64 filters.
- MaxPooling is a 2 × 2 pooling operation with stride 2.
- ReLU activation is applied after each convolutional and dense layer (except the last).

### 2.3. Feedforward Neural Network

A Feedforward Neural Network (FNN) is one of the simplest types of artificial neural networks where information moves in only one direction—forward—from the input layer, through one or more hidden layers, and finally to the output layer. Unlike recurrent neural networks (RNNs) [35], an FNN does not have connections that cycle back; it does not retain the memory of previous inputs, making it well-suited for tasks where temporal dependencies are not critical. Each layer in an FNN consists of a set of neurons that apply a weighted sum of the inputs followed by a non-linear activation function. The model learns these weights during training using a backpropagation algorithm that minimizes a chosen loss function, typically through gradient descent optimization.

In classification tasks, the output layer typically uses a Softmax activation function to provide a probability distribution over classes. FNN is widely used in pattern recognition and function approximation problems due to its simplicity, efficiency, and ability to approximate complex non-linear mappings. The FNN model working principle contains four main steps:

Step 1. Forward Propagation (Prediction Step):

- Input data are passed through the network layer by layer.
- Each neuron computes a weighted sum of its inputs, adds a bias, and applies an activation function.

z = w · x + b(1)

a = f(z)(2)

where

- w: weights vector.
- x: input vector.
- b: bias term.
- f: activation function (e.g., ReLU, sigmoid, tanh).
- a: output of the neuron.
- This continues through all hidden layers to produce the final output.

Step 2. Loss Calculation:

- The network’s predicted output ŷ is compared with the true target y using a loss function based on mean squared error for regression:

(3) $$L=\frac{1}{n}\sum {\left(y-\hat{y}\right)}^{2}\;$$

where $\frac{1}{n}$ is the average of the total error across all *n* samples.

Step 3. Backpropagation (Learning Step):

- The error is propagated backward through the network.
- The gradient of the loss function is computed with respect to each weight using the chain rule.

Step 4. Weight Update:

- Weights are updated using gradient descent based on the formula below:

(4) $$w_{new}=w_{old}-\eta \frac{\partial L}{\partial w}$$

where

*w_old_*: current weight before the update.
*w_new_*_:_ updated weight after applying gradient descent.
η: learning rate: a small positive value that controls how big a step to take in the direction of the gradient.
  - If η is too large → training may overshoot or diverge.
  - If η is too small → training becomes very slow.

- $\frac{\partial L}{\partial w}$: gradient of the loss function L with respect to weights.

This process repeats many iterations (epochs) until the model learns to make accurate predictions.

## 3. Experiment and Result Analysis

The AI models were elaborated and trained by a high-performance workstation equipped with a 13th Gen Intel Core i9-13900 processor running at 2.00 GHz, paired with 64 GB of RAM. It features an NVIDIA GeForce RTX 3070 GPU with 8 GB of VRAM, providing robust support for deep learning, image processing, and GPU-accelerated applications. The system runs a 64-bit operating system on an ×64-based processor architecture, ensuring compatibility with modern software environments. Designed for performance and scalability, it is well-suited for both research and professional applications in fields like machine learning, scientific computing, and multimedia processing.

The grid search method [36] was utilized to select the appropriate hypermeter for the AI models.

The image-based model VGG16 has the following structure:

- The convolutional base of VGG16 (Transfer layer) is used as a fixed feature extractor.
- On top of this, a new classification head was added. This head includes three fully connected layers with 1024 neurons in hidden layer 1 and 512 neurons in hidden layer 2. To enhance training stability and generalization, each of the first two fully connected layers is followed by batch normalization, a Leaky ReLU activation function with a slope of 0.2, and a dropout layer with a rate of 0.4.
- To prevent overfitting, L2 regularization with a small weight decay factor of 10^−5^ is applied to all fully connected layers. This setup helps the model maintain generalization on unseen data.
- The network is trained using the Adam optimizer with an initial learning rate of 10^−5^. A piecewise learning rate schedule is applied, which reduces the learning rate by a factor of 0.5 every 10 epochs.
- Training is configured to run for up to 100 epochs, with a mini-batch size of 8. Validation is performed using an augmented version of the test dataset. Validation frequency is set to every 20 iterations, and early stopping is enabled with the patience of 100 validation checks.

The accelerometer-based model, an FNN, has the following hypermeters:

- Two hidden layers, each containing 20 neurons.
- Maximum number of epoch is 1000.

### 3.1. Image Classification with Visual Geometry Group 16 (VGG16)

**Figure 5:**
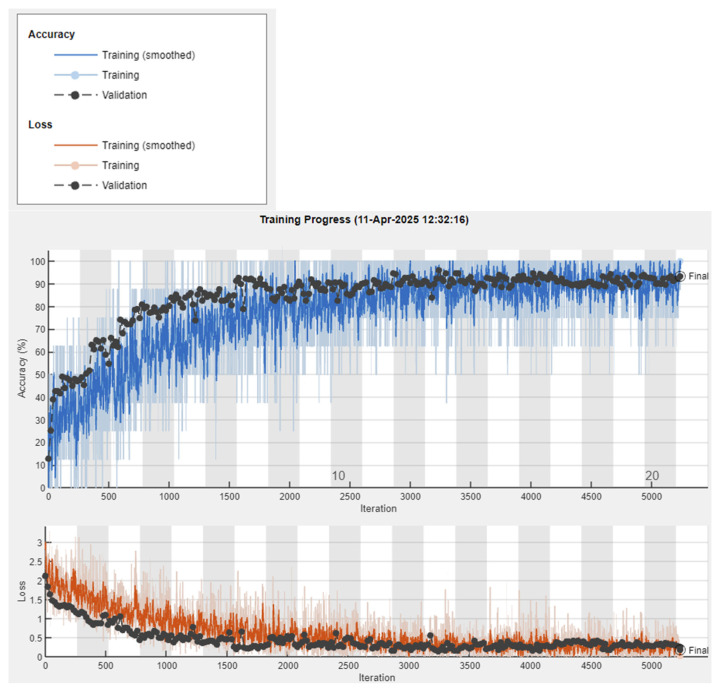
Training process.

As shown in Figure 5, the image classification conducts the validation accuracy of 93.49% with the loss approaching 0 at the epoch of 5240. The total elapsed time is about 28 min.

**Figure 6:**
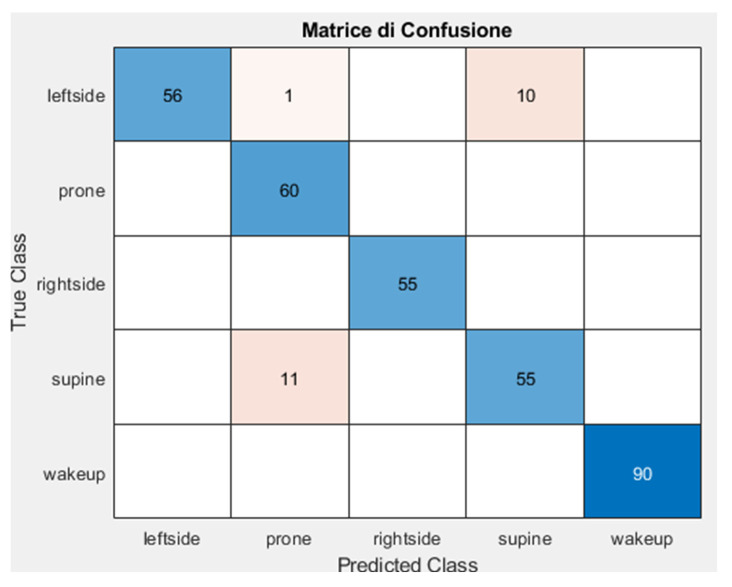
Confusion matrix in the test process of image-based method.

As shown in Figure 6, the confusion matrix demonstrates that the model has good performance overall. There are some mispredictions between prone and supine or supine and left side.

In the test, the model achieved an overall accuracy of 93.49%, indicating strong performance across the entire test set. The high accuracy reflects the model’s ability to generalize well to unseen data after training on augmented sleep posture images.

As reported in Table 1 and Table 2, the right side and wake-up achieved perfect scores with precision, recall, and F1-score = 1.0. These metrics suggest the model is highly confident and consistently correct when predicting these two classes, likely due to well-distinguishable visual features in the images (e.g., clear body orientation or movement cues in wake-up). For the left side, precision is perfect (1.00), meaning when the model predicts “left side”, it is always correct. However, recall is slightly lower (83.58%), indicating that some actual “left side” instances are misclassified (possibly confused with similar poses like “right side” or “supine”). F1-score remains high (91.06%), so performance is still strong.

In terms of supine, this class has the lowest F1-score (83.97%), with precision (84.62%) and recall (83.33%) also the lowest among all classes. This position is the most challenging for the model, which is possibly due to visual similarity with the prone or wake-up poses (e.g., both involve lying flat). Prone has high recall (100%) and solid precision (83.33%), resulting in a strong F1-score (90.91%). The model captures all true prone cases but may occasionally classify other poses like supine as prone.

### 3.2. Accelerometer-Based Sleep Posture Classification

**Figure 7:**
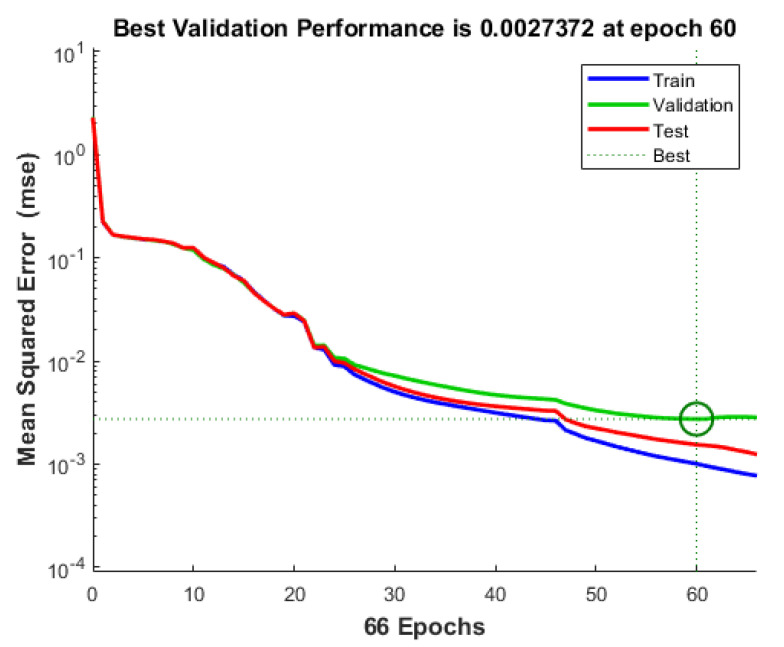
Error behavior during training process.

The accelerometer-based model was trained over 66 epochs out of a possible 1000, completing in approximately 18 s. The least error point is at epoch 60 with a mean squared error (MSE) of 0.0027 as shown in Figure 7. During training, the performance (loss) dropped significantly from an initial 2.3 to nearly zero, indicating effective learning and convergence. The gradient magnitude decreased steadily, showing stable weight updates as the model approached an optimal solution. The adaptive parameter mu was adjusted throughout training, helping the algorithm fine-tune the learning rate, with a large final value signaling training termination due to convergence or early stopping. Validation checks increased during training, suggesting the use of early stopping to prevent overfitting, resulting in a well-optimized and efficient model.

As reported in Table 3, the accelerometer-based model demonstrates exceptional performance, with all classes achieving near-perfect precision, recall, and F1-scores above 99.5%. Precision and recall are balanced across all classes, indicating that the model rarely produces false positives or misses true positives for any sleep position. The slight difference in recall for the Prone class (99.567%) suggests minimal missed instances but remains practically negligible for most applications. This performance highlights the effectiveness of the feedforward network architecture with two hidden layers (20 neurons each) in capturing accelerometer signal patterns corresponding to distinct sleep postures.

With the accelerometer, wake-up is the easiest position to detect because the person is no longer lying in bed, resulting in a clear absence of low-frequency body signals like heartbeat, respiration, or subtle postural vibrations. Unlike lying positions, which exhibit consistent micro-movements due to physiological activity and contact with the bed surface, the wake-up state typically involves a sudden change in motion followed by minimal sensor input, especially if the device is no longer in contact with the body or bed. This stark contrast makes it easily separable in both time-domain and frequency-domain features, leading to highly accurate classification.

**Figure 8:**
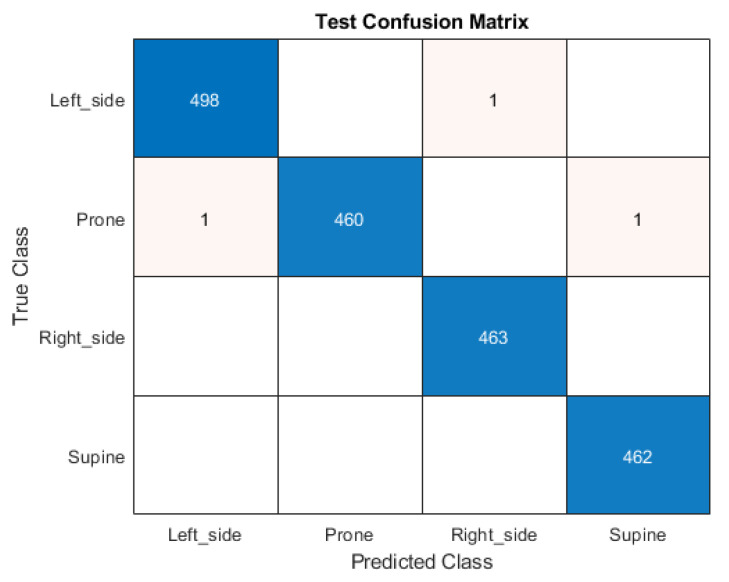
Confusion matrix in test data for accelerometer-based method.

Figure 8 shows the confusion matrix of the accelerometer-based method. Since the wake-up is clearly distinguishable with absolute accuracy, the matrix concentrates on four sleeping postures. There is only one misprediction between the left side and prone; right side and left side; supine and prone. The rest of the test data have the correct predictions.

### 3.3. Comparison of Image-Based Sleep Position Classification and Accelerometer-Based

The accelerometer-based model shows consistently higher precision, recall, and F1-scores across all common sleep positions, with metrics hovering near 100%. This result indicates very stable and reliable classification performance on sensor signals. The image-based model performs excellently on the right side and wake-up classes but shows lower recall and F1-scores for the left side and supine positions. This suggests some difficulty in visually distinguishing certain poses, likely due to similar body orientations or lighting conditions. Accelerometer signals directly capture body movement and orientation, providing more precise, noise-resistant cues for posture recognition than images alone. This is reflected in the near-perfect classification scores. Furthermore, both methods are able to realize the wake-up perfectly with absolute results (Table 4).

As reported in Table 5, the Image-Based Model has significantly larger input data (343.15 MB) compared to the Accelerometer Model (22.3 MB) because image data are usually high-dimensional (224 × 224 RGB images), while accelerometer data are a 1D time series with fewer features. Moreover, the Image-Based Model took 1680 s (28 min) to train, while the Accelerometer Model completed training in just 18 s due to higher computational load with more layers and parameters in image models. The pros and cons of these two approaches are reported in Table 6.

## 4. Limits and Future Work

Despite the high accuracy achieved by both methods, this study has some limitations. Although the accelerometer-based approach is highly precise, it is sensitive to external vibrations and noise, which may reduce its effectiveness in less controlled or dynamic environments. The image-based method showed lower and more variable accuracy, particularly for prone and supine positions, likely due to variations in lighting, occlusions, and subject posture. In addition, privacy concerns associated with camera-based monitoring and the higher computational demands of the image-based model further restrict its practical deployment in healthcare settings.

Future work could focus more on the test condition with noise for accelerometer data and investigate multimodal approaches that fuse sensor and image information to enhance classification accuracy and reliability. The datasets can be expanded to include more diverse data and more sleep scenarios would also improve model generalization. Developing lightweight and efficient models suitable for wearable or edge devices could facilitate real-time monitoring, while privacy-preserving techniques such as on-device processing could address ethical concerns around image use. Lastly, long-term studies in natural sleep environments and integration of sleep position recognition with clinical applications like sleep disorder diagnosis represent promising directions for advancing this research.

## 5. Conclusions

This paper presented a comparative analysis of two approaches for sleep position recognition: an image-based method using a VGG-16 deep convolutional neural network and an accelerometer-based method utilizing a feedforward neural network. The results demonstrated that both methods achieved high classification performance across five sleep positions (supine, prone, left side, right side, and wake-up). Notably, the accelerometer-based model outperformed the image-based approach, reaching an overall accuracy exceeding 99.8% and exhibiting strong precision, recall, and F1-scores across all classes. In contrast, the image-based model, while still effective, achieved slightly lower accuracy (approximately 93.5%) and showed more variability across classes—particularly for the prone and supine positions. These findings highlight the robustness and efficiency of sensor-based classification with faster training time and lighter datasets, especially in privacy-sensitive environments such as healthcare and sleep monitoring. On the other hand, the accelerometer method requires no external vibration interference to guarantee the best accuracy since the noise can influence model performance. The comparison in the training time, data size, and specific characteristics provides a comprehensive evaluation of both methods, analyzing the advantages and disadvantages of each system.

## Figures and Tables

**Table 1:** VGG 16 Components.

Layer Type	Configuration
Input	224 × 224 RGB image (three channels)
Conv Block 1	2 × Conv3-64 + MaxPooling
Conv Block 2	2 × Conv3-128 + MaxPooling
Conv Block 3	3 × Conv3-256 + MaxPooling
Conv Block 4	3 × Conv3-512 + MaxPooling
Conv Block 5	3 × Conv3-512 + MaxPooling
Fully Connected 1	Dense 4096 + ReLU
Fully Connected 2	Dense 4096 + ReLU
Fully Connected 3 (Output Layer)	Dense 5 + Softmax

**Table 2:** Image classification results of test process.

Class	Precision	Recall	F1-Score
Left Side	1.00	0.83582	0.91057
Right Side	1.00	1.00000	1.00000
Supine	0.84615	0.83333	0.83969
Prone	0.83333	1.00000	0.90909
Wake-up	1.00	1.00000	1.00000
Overall Accuracy: 93.49%			

**Table 3:** Test results of accelerometer-based approach.

Class	Precision (%)	Recall (%)	F1-Score (%)
Left Side	99.8	99.8	99.8
Prone	100	99.567	99.783
Right Side	99.784	100	99.892
Supine	99.784	100	99.892
Wake up	100	100	100
Overall accuracy: 99.84%			

**Table 4:** Metric comparison.

Class	Image-Based Model (Precision/Recall/F1)	Accelerometer Model (Precision/Recall/F1%)
Left Side	100/83.58/91.06	99.8/99.8/99.8
Prone	83.33/100/90.91	100/99.57/99.78
Right Side	100/100/100	99.78/100/99.89
Supine	84.62/83.33/83.97	99.78/100/99.89
Wake-up	100/100/100	100/100/100

**Table 5:** Data size and time comparison.

Model	Data Size (MB)	Training Time (s)
Image-Based Model	343.15 MB	1680 s
Accelerometer Model	22.3 MB	18 s

**Table 6:** Characteristics Comparison.

Aspect	Image-Based Model	Accelerometer-Based Model
Data Size	Larger, requires high storage for RGB image sequences	Lightweight, low storage due to compact time-series data
Training Time	Slower, requires high computational resources (GPU)	Faster, it can be trained quickly due to the light dataset
Power Efficiency	High power consumption due to intensive computation and GPU use	Low-power devices like microcontrollers are sufficient
Privacy	Captures identifiable visual data, raising privacy concerns	Non-invasive and private, with no visual identity exposure
Accuracy	High, especially in controlled environments with clear visuals	Good accuracy using motion data; may drop if motion is minimal
Feature Engineering	CNNs automatically learn features from data	Pre-feature extraction (e.g., sum, std, max, spikes) needed
Environmental Sensitivity	Sensitive to lighting, occlusions, and camera angles	Sensitive to external vibrations and sensor placement
Hardware Requirements	Requires camera(s), stable lighting, and good angle setup	Requires microcontroller, accelerometer sensor, and cabling
Deployment Environment	Best in controlled, well-lit rooms with minimal occlusion	Works in dark or varied lighting, but needs vibration control

## Data Availability

Data are available from the corresponding author upon reasonable request.
